# Percutaneous endoscopic lumbar discectomy for lumbar disc herniation as day surgery – short-term clinical results of 235 consecutive cases

**DOI:** 10.1097/MD.0000000000018064

**Published:** 2019-12-10

**Authors:** Jian Cao, Wenzhou Huang, Tianlong Wu, JingYu Jia, Xigao Cheng

**Affiliations:** Department of Orthopedic Surgery, The Second Affiliated Hospital of Nanchang University,.

**Keywords:** percutaneous endoscopic lumbar discectomy, lumbar disc herniation, day surgery

## Abstract

Many studies have reported the good outcomes of percutaneous endoscopic lumbar discectomy (PELD) for the treatment of lumbar disc herniation (LDH). However, the majority of published studies on PELD showed an average hospital stay of 2 to 5 days. Thus, the purpose of this retrospective study was to evaluate and compare the clinical outcomes of patients undergoing PELD for LDH as day surgery with the outcomes of patients managed as inpatients.

A total of 402 patients who underwent PELD for single-level LDH were included. The visual analog scale score (VAS) for leg and back pain, Oswestry Disability Index (ODI) score, and Macnab criteria were evaluated preoperatively and at 2 years postoperatively (final follow-up). Operation time, duration of hospital stay, cost, postoperative complications, and the rates of and reasons for delayed discharge and readmission were recorded and analyzed.

The mean operative time was 45.8 ± 8.4 minutes in the PELD-A (nonday surgery mode) group and 41.3 ± 8.7 minutes in the PELD-D (day surgery mode) group (*P* = .63). The average duration of hospital stay was 2.8 ± 1.1 days in the PELD-A group and 3.2 ± 0.9 hours in the PELD-D group (*P* < .001). The average hospitalization expenses of the PELD-A and PELD-D groups were 28,090 ± 286 RMB and 24,356 ± 126 RMB (*P* = .03), respectively. In both groups, the mean VAS and ODI scores improved significantly postoperatively compared with the preoperative scores. The satisfactory result rate was 89.8% in the PELD-D group and 91.0% in the PELD-A group, without a significant difference (*P* = .68). The delayed discharge rate in the PELD-A and PELD-D groups was 8.20% and 8.43%, respectively (*P* = .93). The main reasons for delayed discharge were dysesthesia, neurologic deficit, nausea, headache and residential distance from the hospital. The overall readmission rates were 5.99% and 5.53% in the PELD-A and PELD-D groups, respectively (*P* = .85). The most common reasons for readmission were reherniation, sequestered herniation and pain.

In conclusion, PELD is safe and effective for the treatment of LDH and can reduce medical costs as day surgery, and it thus warrants increased attention.

## 1 Introduction

Lumbar disc herniation (LDH) is a leading cause of low back pain and radiculopathy and is considered the most common ailment requiring lumbar spinal surgery, which often occurs at the L4/5 or L5/S1 level.^[1,2]^ If the patient's symptoms are not relieved after 6 weeks of conservative treatment, an operation is often needed.^[3]^ The advantages of percutaneous endoscopic lumbar discectomy (PELD) include a 7 mm skin incision, local anesthesia, light tissue damage, rapid recovery and favorable outcomes.^[4–7]^ Additionally, with the development of intervertebral foraminoplasty, any type of LDH could be resolved by PELD.^[8]^ An increasing number of people turn to PELD first to address disc herniation. Although some experts believe that PELD can treat disc herniation as day surgery, the majority of published studies on PELD still show an average hospital stay of 2 to 5 days.^[9–14]^ The purpose of this study was to evaluate and compare the clinical outcomes of patients undergoing PELD for LDH as day surgery with the outcomes of patients managed as inpatients.

## 2 Materials and methods

The study was approved by the medical ethical council of the authors’ institution. All patients agreed to sign informed consent at study entry. From Aug 2013 to Oct 2015, 183 patients (PELD-A Group) received PELD surgery as inpatients, while a total of 261 patients (PELD-D Group) received PELD surgery and were discharged on the day of surgery from Nov 2015 to Jan 2017. MR neurography (MRN), magnetic resonance imaging (MRI), computed tomography (CT) scan, and fluoroscopy examinations were performed to confirm the diagnosis of LDH.

The inclusion criteria were as follows:

(a) single-level LDH (L4/5, L5/S1) confirmed by computed tomography scan and magnetic resonance imaging with corresponding symptoms and signs, and
(b) failure of conservative therapy after 6 weeks.

The following exclusion criteria were applied:

(a) nonadherence to the above inclusion criteria,
(b) segmental instability or foraminal stenosis,
(c) lumbar spondylolisthesis,
(d) motor weakness,
(e) previous lumbar surgery at the affected level, and
(f) spinal tumor or infection.

### 2.1 Surgery procedure

**Figure 1:**
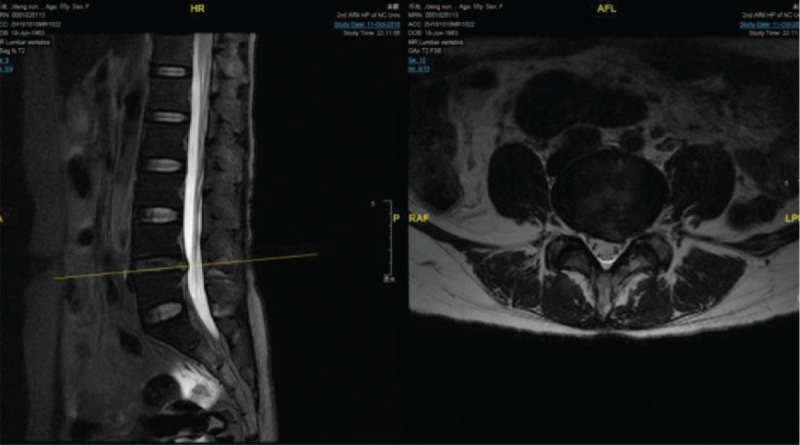
Preoperative magnetic resonance imaging (MRI) shows lumbar disc herniation at L4-L5.

**Figure 2:**
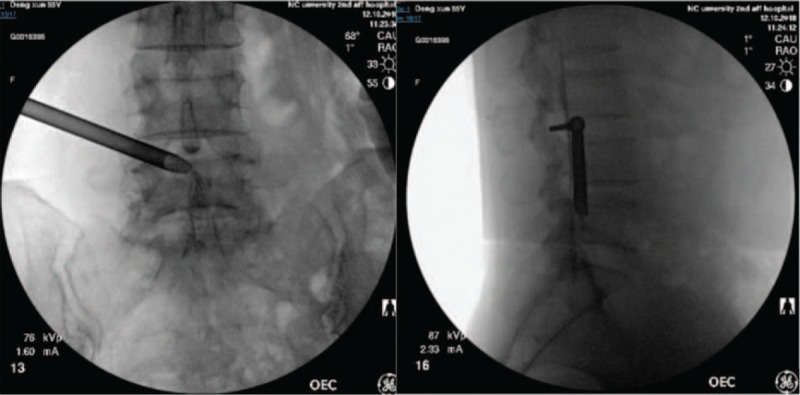
The working channels reaching the operation area at L4-L5.

**Figure 3:**
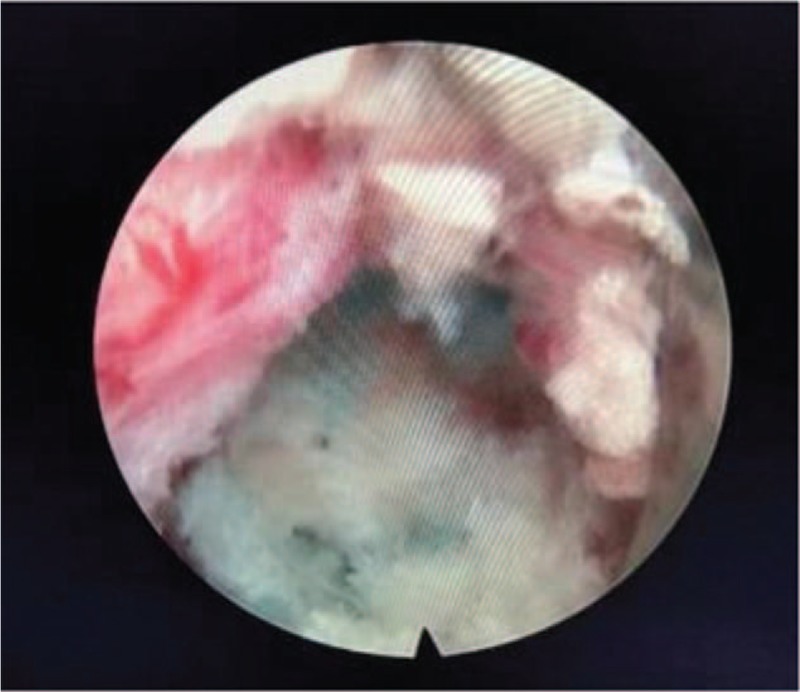
At the final step, the anatomical details are well demonstrated including the decompressed NR and the maternal disc.

All operations were performed by 2 surgeons who had many years of experience in the PELD technique. Patients were given an analgesic (flurbiprofen axetil 50 mg) 30 minutes before surgery. All patients enrolled in the study were subjected to PELD. Patients were positioned in the lateral decubitus position. The surgical area was disinfected, and local anesthesia was initially performed at the entry point of the needle, approximately 10 to 11 cm away from the midline. Then, an 18-G needle was introduced to anesthetize the path with 8 to 10 ml 1% lidocaine. When reaching the superior articular process, 2 to 3 ml 1% lidocaine was used to anesthetize the facet joint. Lidocaine was added intraoperatively, if necessary. The following procedures were performed according to the standard percutaneous transforaminal endoscopic spine system (TESSYS) (5.5, 6.5, 7.5 mm, Joimax System) by two experienced surgeons.^[3]^ At the end of the surgery, the dural sac and nerve-root were freely mobilized (Figs. 1–3). The skin was closed using 3-0 nylon sutures. If the postoperative evaluation of VAS was less than 3 points, patients were discharged 2 to 3 hours after surgery. All the patients were required to maintain bed rest for 4 weeks.

### 2.2 Data collection

Telephone and clinical follow-ups were evaluated according to the following criteria:

(a) functional status was assessed by the Oswestry Disability Index (ODI (0–100%)) at 2 years postoperatively;
(b) level of leg pain was assessed according to a 10-point VAS;
(c) back pain was rated according to a 10-point VAS; and
(d) surgical effectiveness was rated by the Macnab criteria at the final follow-up.

Additionally, operation time, duration of hospital stay, cost, postoperative complications, the rates of and reasons for delayed discharge and readmission were recorded and analyzed.

### 2.3 Statistical analyses

Collected data were analyzed with the use of SPSS 22.0 statistical software (SPSS, Inc., Chicago, IL). Quantitative data were shown as the mean ± standard deviation, and qualitative data were expressed as the frequency (%). The normality of the data was analyzed. The Mann–Whitney U test was utilized for intergroup data comparison. The Wilcoxon test was used for the data analysis within the PELD-A and PELD-D groups. Intergroup Macnab outcomes and delayed discharge and readmission factors were compared with a x^2^ test on a four-fold table. *P* < .05 indicated that the difference was statistically significant.

## 3 Results

Forty-two patients were not followed up for various reasons (e.g., immigration abroad, car accidents, death). According to the study criteria, there were 167 patients in the PELD-A (nonday surgery mode) group, with 92 males (55.1%) and 75 females (44.9%). The average age of this group was 44.5 ± 13.8 years (range 14–83 years). There were 235 patients in the PELD-D (day surgery mode) group, with 134 males (57.0%) and 101 females (43.0%). The average age of this group was 45.7 ± 11.7 years (range 13–82 years). The operation time in the PELD-A group was similar to that of the PELD-D group. The mean operative time was 45.8 ± 8.4 minutes in the PELD-A group and 41.3 ± 8.7 minutes in the PELD-D group (*P* = .63). The blood loss of the 2 groups of patients was negligible and had no significant clinical influence. The average duration of stay at the hospital was 2.8 ± 1.1 days in the PELD-A group and 3.2 ± 0.9 hours in the PELD-D group (*P* < .001). The average hospitalization expenses for the PELD-A and PELD-D groups were 28,090 ± 286 RMB and 24,356 ± 126 RMB (*P* = .03), respectively. There were no significant differences between 2 groups in gender, age, surgical level and operation time. All included patients successfully completed PELD without transfer to open surgeries. The basic characteristics of all the patients are demonstrated in Table 1.

**Table 1:**
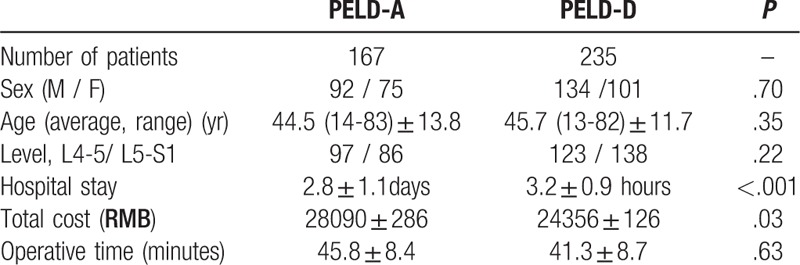
General information of patients in the 2 groups.

In the PELD-D group, 213 patients were discharged after day surgery, with 22 patients having a delayed discharge from the hospital. A total of 15 patients in the PELD-A group and 14 patients in the PELD-D group had surgical complications. The rate of surgical complication was 8.20% in the PELD-A group and 5.36% in the PELD-D group, with no significant difference in the complication rate between the two groups (*P* = .23). A total of 7 patients in the PELD-A and 8 patients in the PELD-D group experienced postoperative dysesthesia. The postoperative dysesthesia was relieved, and the patients gradually recovered after 3 months of oral Mecobalamin or other conservative treatment such as functional exercise.^[15]^ Motor deficits occurred in 3 patients in the PELD-A group and 1 patient in the PELD-D group. Two cases of motor deficits in the PELD-A group were permanent, with foot and toe weakness or foot drop. The remaining two cases of motor deficits in the two group were transient and recovered completely after rehabilitation. (Table 2)

**Table 2:**
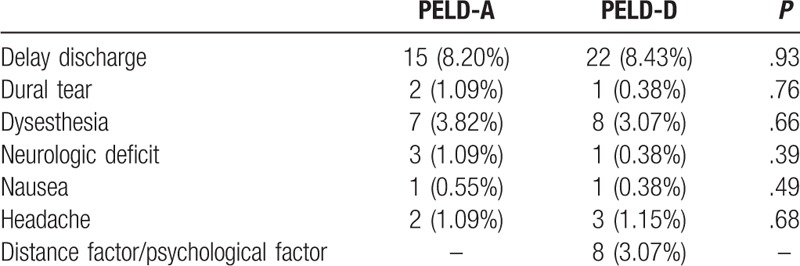
Statistical analysis of delay discharge factors.

Patients with a delayed discharge for “other” reasons included patients who lived far away from our institution and patients reporting psychological factors, nausea, dural tear and headache (Table 2). We recommended these patients stay in the hospital to observe the effect of treatment. There were no episodes of hemorrhage and no intraoperative vascular injuries.

The overall readmission rate was 5.99% in the PELD-A group and 5.53% in the PELD-D group. There was no significant difference in the readmission rate between the two groups (*P* = .85). The most common reasons for readmission were reherniation, sequestered herniation and neuralgia. There were no reports of wound infection in either of the two groups (Table 3). We performed MRI in patients with gradually worsening pain and found residual disc tissue. These patients underwent reoperation with the same technique. Glucocorticoids and mannitol were used to treat postoperative neuralgia^[16,17]^ (Table 3).

**Table 3:**
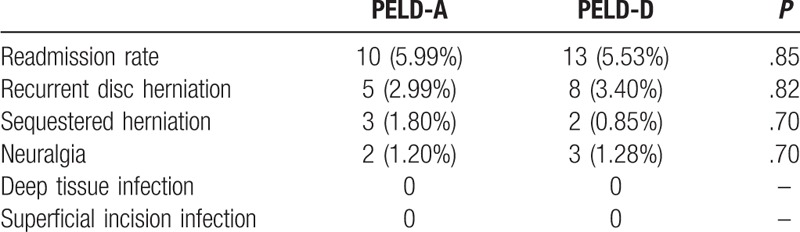
Statistical analysis of readmission factors.

Based on the Macnab criteria, the surgical outcomes at 2 years postoperatively were as follows:

1. PELD-A group—Out of 167 patients, 75 had excellent results, 77 had good results, 8 had fair results, and 7 had poor results.
2. PELD-D group—Out of 235 patients, 106 had excellent results, 105 had good results, 14 had fair results, and 10 had poor results.

The satisfactory result rate was 91.0% in the PELD-A group and 89.8% in the PELD-D group, without a significant difference (*P* = .68). An average improvement in leg pain of 6.58 points on the VAS was recorded (from 7.46 ± 1.1 preoperatively to 0.88 ± 2.4 at 2 years postoperatively) in the PELD-A group (*P* = .01). Similarly, an average improvement in back pain of 2.89 points on the VAS was recorded (from 4.38 ± 1.2 preoperatively to 1.49 ± 1.2 at 2 years postoperatively) in the PELD-A group (*P* = .02). In the PELD-D group, the VAS score for leg pain (VAS-L) ranged from 7.12 ± 1.3 to 0.83 ± 2.1 at 2 years postoperatively (*P* = .02), and the VAS score for back pain (VAS-B) ranged from 4.41 ± 1.1 to 1.12 ± 1.3 at 2 years postoperatively (*P* = .03). The ODI score at the final follow-up was 11.86 ± 5.8 in the PELD-A group and 7.39 ± 5.3 in the PELD-D group (*P* = .56). No significant differences in the VAS pain ratings and ODI scores were found between the two groups after surgery (Table 4). Moreover, no significant differences in the Macnab (*P* = .68) outcomes was found between the 2 groups (Table 5).

**Table 4:**
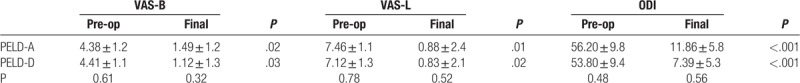
Comparison of VAS-B, VAS-L and ODI scores between 2 groups.

**Table 5:**
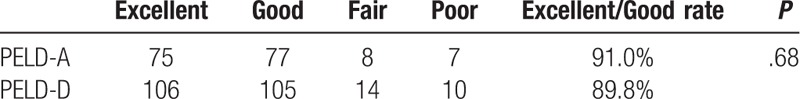
Comparison of MacNab evaluation in 2 groups.

## 4 Discussion

Day surgery is defined as an operation/procedure that allows patients to be discharged on the same working day, excluding an office or outpatient operation/procedure.^[18]^ Day surgery, which is associated with short times, low risk of infection, fast recovery and low cost, is becoming increasingly popular with clinicians and patients.^[19]^ For patients, day surgery can reduce waiting times and hospital stays. Patients spend less money to undergo the same surgery. With limited healthcare recourses in society, day surgery can increase the patient turnover rate, shorten hospital bed days, reduce the insurance burden and increase hospital profits.^[19]^ Due to these advantages, day surgery has developed significantly in the USA and UK. For example, a day surgery center in America performed 3000 operations in the first year of its establishment.^[19]^ Steger et al reported that 8.8% of patients who underwent endoscopic inguinal hernia repair as day surgery had hematomas and 2.9% of all patients had hernia recurrence. No infections occurred after endoscopic inguinal hernia repair.^[20]^ Satisfaction and acceptance by the patients were very high. Patients with wound infections after surgery usually had poor outcomes and even death.^[21]^ Alexis Laurent et al found that only one patient (4.2%) was readmitted due to a wound infection.^[22]^ A recent systematic review showed that the surgical site infection rate was 1% to 2% following day surgery, regardless of the surgical procedure.^[23]^

Traditional open surgery has become the most popular choice for LDH.^[24]^ However, the many shortcomings and the experience of discomfort can negate the beneficial effects of the surgery itself.^[25]^ With the development of minimally invasive technology and related devices, remarkable progress has been made in LDH. Compared to open surgery, PELD requires only a 7 mm skin incision. Due to the use of local anesthesia, the patient and surgeon can communicate during the surgery to easily confirm efficacy.^[11,26]^ In addition, the symptoms can be relieved immediately after surgery. Thus, PELD for day surgery is feasible. However, the majority of published studies on PELD still show an average hospital stay of 2 to 5 days^[9–14]^, and patients need to stay in the hospital, which increases the risk of nosocomial infection. After years of practice, the technology has matured, and the feedback from patients has given us the courage to perform day surgery. This is the first study to report the outcomes of PELD as day surgery to treat LDH.

In our series, we reviewed 2 cohorts of day and nonday procedures from a single institution. The aim was to compare the outcomes between the 2 procedures. A previous study recommended evaluating the effect of nerve root decompression at 1 to 2 years after the operation, which was considered to be when the healing process was completed.^[27]^ Thus, the follow-up of our study is reasonable. Both groups exhibited significant clinical improvement in pain and functional parameters at the last follow-up. At 2 years after surgery, there were no significant differences in the back and leg pain scores between the two groups. The PELD-D group appeared to have VAS and ODI scores comparable to those of the PELD-A group at the last follow-up. The satisfactory result rate of 89.8% in the PELD-D group is comparable to the outcome of 91.0% in the PELD-A group and the outcome of 88.2% in a restricted endoscopic series by Kambin et al.^[28]^ Based on these results, PELD as day surgery can achieve equal effects as PELD requiring hospitalization in the treatment of LDH.

Traditional open surgery is an important choice for LDH. A previous study showed that the outcome of PELD was comparable to that of conventional open surgery.^[29]^ According to our final follow-up results, including the VAS-L, VAS-B, ODI and Macnab, the results of PELD were comparable to those observed in published series on PELD (nonday surgery mode) (Table 6).

**Table 6:**
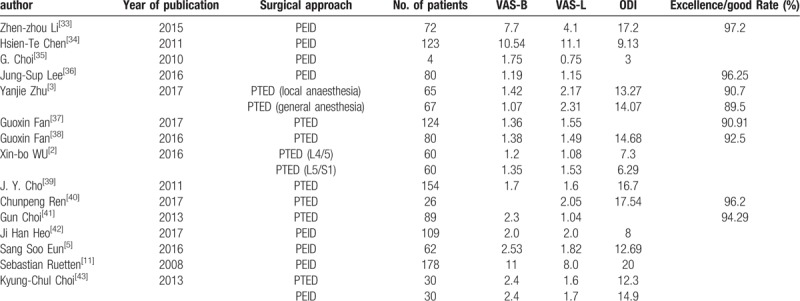
Comparison of the final follow-up results with published study's (non-day surgery mode).

The probability of complications in the PELD-A and PELD-D groups was 8.20% and 5.36%, respectively, which was similar to the reported results.^[13,30]^ These factors may delay discharge. Postoperative dysesthesia (POD) was the main surgical complication. A total of 7 patients had postoperative dysesthesia in the PELD-A group, whereas 8 patients had POD in the PELD-D group. Chi et al pointed out that heat transfer from the radio frequency coagulator to the nerve structure was the main cause of postoperative dysesthesia.^[28]^ The mechanical compression of dorsal root ganglia by the cannula in the epidural space could be another cause of dysesthesia.^[31]^

Hospital observation is necessary if the patient has severe leg pain, headache, nausea, chest tightness, dural tear, or other complications, such as posterior neck pain, abdominal pain (especially retroperitoneal hematoma), intraoperative vascular injuries and bladder and/or rectal disturbance after surgery, which may lead to delayed discharge. We found that the main reason for readmission was hernia recurrence. The rate of recurrent disc herniations in the PELD-A and PELD-D groups was 2.99% and 3.40%, respectively, and showed no significant difference (*P* = .82). The rates correspond to data in the literature.^[13]^ Schaffer et al reported that the most common causes of hernia recurrence were lateral recess stenosis, sequestered herniation and improper placement of the working instruments^[32]^, while we found that some recurrences of intervertebral disc herniation can be attributed to weight-bearing activity within 1 month after operation. In our study, no patient showed infections after PELD as day surgery.

The effect of day surgery is indistinguishable from that of nonday surgery. Therefore, the length of hospitalization was not related to the outcome of surgery. The main factors affecting the outcome of surgery may be related to the operator's operation. However, a shortcoming of this study is the small number of cases. Additionally, due to family culture and patient psychological factors, some patients chose to delay discharge after surgery, which affected the authenticity of the data. The patient's pain assessment was determined by the patient's institution and may not be very accurate.

## 5 Conclusions

PELD for LDH as day surgery has the same safety and effectiveness as the nonday surgery mode while significantly reducing the average hospital stay and hospitalization costs for patients. Additionally, it enables hospitals to use limited health resources more efficiently. Therefore, it warrants increased popularity among surgeons.

## Acknowledgments

We thank Drs. Xi Xiong and Hui Wu in the study management, including the systemic examination, follow-up visits, and advice regarding the clinical approach. Thanks to Hui Zhang for helping us with data analysis.

## Author contributions

**Writing – review & editing:** Xigao Cheng.
